# Impact of presurgical breast MRI in a district general hospital

**DOI:** 10.1186/bcr2987

**Published:** 2011-11-04

**Authors:** A Newland, M Dumba, S Flais

**Affiliations:** 1 Ealing Hospital, London, UK

## Introduction

Routine MRI in the assessment of lobular carcinoma, invasive ductal carcinoma in dense breasts, patients younger than 35 or radioclinical discordance at the time of cancer diagnosis was introduced in the symptomatic breast unit of Ealing Hospital in 2009. We analyse the impact of this new pathway on our local practice.

## Methods

All patients with a new diagnosis of breast cancer in two periods of 18 months, before and after the introduction of MRI, were reviewed. Mastectomy rates were compared between the two groups and the role of MRI in this change is discussed. The cost of the new pathway is reviewed, including the cost of additional imaging and biopsies generated by MRI.

## Results

The mastectomy rate increased from 25% between 1 January 2006 and 30 June 2007, to 40% between 1 January 2010 and 30 June 2011. Other factors (patient group, cancer grade, multidisciplinary team decision-making process) were unchanged. The cost of the presurgical assessment of patients undergoing MRI has increased significantly.

## Conclusion

Presurgical MRI has changed the first-line treatment offered by our team, but at a significant cost, especially for the radiology department. The local impact on overall survival and event-free survival is still unknown.

